# Correlating the oral swab microbial community with milk production metrics in Holstein dairy cows

**DOI:** 10.1128/msphere.00167-25

**Published:** 2025-05-14

**Authors:** Joseph H. Skarlupka, Madison S. Cox, Andrew J. Steinberger, Dino L. Sbardellati, Andrew J. Scheftgen, Ibrahim Zuniga-Chaves, Eric Paget, Charles Skadron, Nithya Attipetty, Jennifer C. McClure, Derek M. Bickhart, Garret Suen

**Affiliations:** 1 Microbiology Doctoral Training Program, University of Wisconsin—Madison 5228, Madison, Wisconsin, USA; 2 Department of Bacteriology, University of Wisconsin—Madison 5228, Madison, Wisconsin, USA; 3 Department of Allergy and Infectious Disease, University of Washington School of Medicine 12353 , Seattle, Washington, USA; 4 Microbiology Graduate Group, University of California—Davis 8789, Davis, California, USA; 5 USDA Dairy Forage Research Center, Madison, Wisconsin, USA; 6 Hendrix Genetics, Boxmeer, the Netherlands; NC State University, Raleigh, North Carolina, USA

**Keywords:** oral swab, rumen community, oral community, milk production efficiency, milk yield, next-generation sequencing

## Abstract

Oral swabs of dairy cows have been suggested as a proxy for direct ruminal sampling, and this approach can identify the presence of up to 70% of the rumen microbial community. Here, we further extend the utility of this approach by correlating the bacterial community of swabs collected from 226 dairy cows on a research farm in Wisconsin, USA, with average milk yield and days in milk, two phenotypes previously associated with differences in the ruminal microbiome. We then obtained milk production efficiency data for a subset of these animals (gross feed efficiency [GFE] and residual feed intake [RFI]) and correlated these metrics against their associated microbial data. We found that when using the oral swabs, we could identify correlations between bacterial genera and days in milk (*P* < 0.05). We further show that the ruminal microbiota was associated with average milk yield and days in milk for animals in their first lactation. Differential abundance testing identified amplicon sequence variants (ASVs) associated with these metrics (*P* < 0.05). Our comparison of bacterial communities between high and low efficiency groups, as determined by GFE and RFI, identified a significant difference in Shannon’s diversity in second lactation cows (*P* < 0.05). We also found that RFI was significantly correlated with the bacterial community in second lactation animals (*P* < 0.05). Differential abundance analysis identified multiple oral- and rumen-associated ASVs correlated with GFE and RFI (*P* < 0.05). This study further establishes the utility of oral swabs as a ruminal proxy.

**IMPORTANCE:**

Improving milk production efficiency is a key goal in the dairy industry and is traditionally pursued through genetic selection, diet optimization, and herd management practices. The ruminal microbiome, essential for digesting feed, has been linked to milk production efficiency, suggesting that microbiome modulation could improve efficiency. However, the integration of rumen microbiology into current management practices is hampered by the difficulty of large-scale rumen sampling, as proxies like fecal samples do not accurately reflect the ruminal microbiota. Traditional methods, like cannulation and stomach tubing, are labor-intensive and impractical for extensive sampling. Our research demonstrates the potential use of oral swabs as a scalable, effective method for characterizing the microbiome and its associations with milk production metrics, recapitulating established associations obtained through traditional ruminal sampling methods.

## INTRODUCTION

The ruminal microbial community in dairy cows is responsible for providing nutrients to the animal from otherwise indigestible feed (1, 2). As such, previous work has demonstrated that the ruminal microbiome is directly linked to the animal’s milk production efficiency (MPE) (3, 4). Broadly, MPE is a measurement of a cow’s ability to convert calories from feed into calories of milk. MPE can be described through a variety of metrics, including gross feed efficiency (GFE) and residual feed intake (RFI). Characterizing the rumen microbiome of a herd can provide producers with a wealth of information that can be used to improve overall production. For example, incorporating rumen microbial data into farm management has the potential to improve production via early detection of metabolic disorders like subacute ruminal acidosis, selection for microbial characteristics that improve MPE via breeding efforts, application of supplements to modulate the community, or decrease methane production (5–10).

Studies linking the rumen microbiome to milk production usually employ direct sampling of the rumen. These methods include collecting contents via a tube inserted through the mouth (stomach tubing), using a needle to collect contents through the wall of the rumen (rumenocentesis), and collection via cannulas surgically installed on the side of the animal (4, 11–13). For example, Jami et al. (11) used stomach tubing to identify bacterial genera that were significantly and highly correlated with various milk production metrics in a cohort of lactating cows. Similarly, Jewell et al. (4) also described changes in diversity and composition over time, alongside differences in composition between cannulated cows of differing MPE.

It is important to note that the application of these methods for collecting rumen contents directly from the cow makes sampling large herds largely infeasible (8). Other approaches, such as direct fecal sampling, which has been used in humans and other animal systems to characterize the gut microbial community (14–16), are not representative of the rumen, as the environments of the rumen and lower GI tract are vastly different, resulting in differences in microbial community structure (9, 17–19). These differences make it difficult to relate changes in the rumen microbial community with milk production efficiency. Recently, oral swabs have been proposed as a method for collecting rumen contents regurgitated during rumination, and previous work has demonstrated the utility of this approach for capturing the ruminal microbial community (8, 9). Importantly, this approach is noninvasive and can be easily and rapidly collected, making it ideal for “convenience” sampling at the herd level. Previous work by our group has shown that oral swabs can capture the presence of upwards of 70% of the ruminal bacterial community (20).

Researchers are only beginning to use oral swabs as a proxy to associate the rumen microbiome with traits of interest, such as feed intake (21). However, widespread adoption of this approach requires standardization of methods and continued demonstrations of this method at a large scale to confirm that oral swabs can recapitulate known associations obtained using “gold standard” ruminal sampling techniques, such as via a cannula or stomach tubing. To address this, we collected oral swabs from more than 200 cows that were in their first, second, or third lactations on a large research farm in south-central Wisconsin, USA. We then generated microbiome data from these swabs and paired them with host milk production metrics. We then identified correlations between the structure of the bacterial community and the herd’s production metrics. We hypothesized that alpha diversity metrics would not differ significantly between animals, whereas beta diversity metrics would show significant associations with production metrics, mirroring previous studies that directly collected rumen samples. Additionally, we expect individual bacterial community members to be differentially abundant between high and low milk-producing animals.

## RESULTS

### Sequencing and cleanup

Swab samples from a total of 226 cows were sequenced, resulting in 7,081,688 raw reads and averaging 31,334 reads per sample (±43,260). Read counts ranged from 5,059 to 352,630 reads. After cleanup, a total of 5,913,320 reads remained, with an average of 26,165 reads (±36,273), and a range of 4,179–295,963 reads per sample. Rarefaction analysis of the entire data set showed that 7,000 sequences were required for alpha diversity analysis, and thus, a total of 18 samples with <7,000 sequences were removed from our analysis.

### The structure of the bacterial swab community is associated with days in milk and milk yield

A Wilcoxon rank sum pairwise test of alpha diversity scores between groups split by parity and stage of lactation indicated no significant differences between groups (*P* > 0.05; Fig. S1). To visualize the differences in overall community structure between high and low milk yield production groups, a principal component analysis (PCA) plot using Aitchison distances of the centered log-ratio (CLR) transformed abundances was generated (Fig. S2). These plots show an overlap between groups, and a PERMANOVA indicated that there were no significant differences between groups of animals based on tier of milk yields (*P* > 0.05).

**Fig 1:**
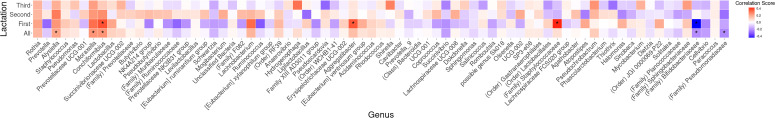
Spearman’s correlation heatmap of the top 75 genera against days in milk. The relative abundances of the top 75 genera with >0.1% relative sequence abundance in at least one animal and over 50% prevalence in all animals were correlated against days in milk. Correlations were determined for all animals combined and for the individual parities. The darkness of the color indicates the strength of the negative (purple) or positive (red) correlation score, with an asterisk indicating a significant relationship between days in milk and the relative abundance with false discovery rate correction for multiple tests (*P* < 0.05).

Spearman’s rank correlation was used to determine genera associated with days in milk (DIM) or milk yield. When comparing all animals, regardless of parity, the genera *Alysiella*, *Moraxella*, *Conchiformibius*, and *Lactobacillus*, along with the families *Sphingobacteriaceae* and *Pseudomonadaceae*, were significantly correlated with DIM (Fig. 1). In the first lactation, members of the genus *Conchiformibius* and the families *Aggregatibacter, Staphylococcaceae*, and *Sphingobacteriaceae* were significantly correlated with DIM (Fig. 1). There were no significant correlations identified in second and third lactation animals (*P* > 0.05). When correlating relative abundances of genera with milk yield, there were no significant correlations identified (*P* > 0.05; Fig. S3).

**Fig 2:**
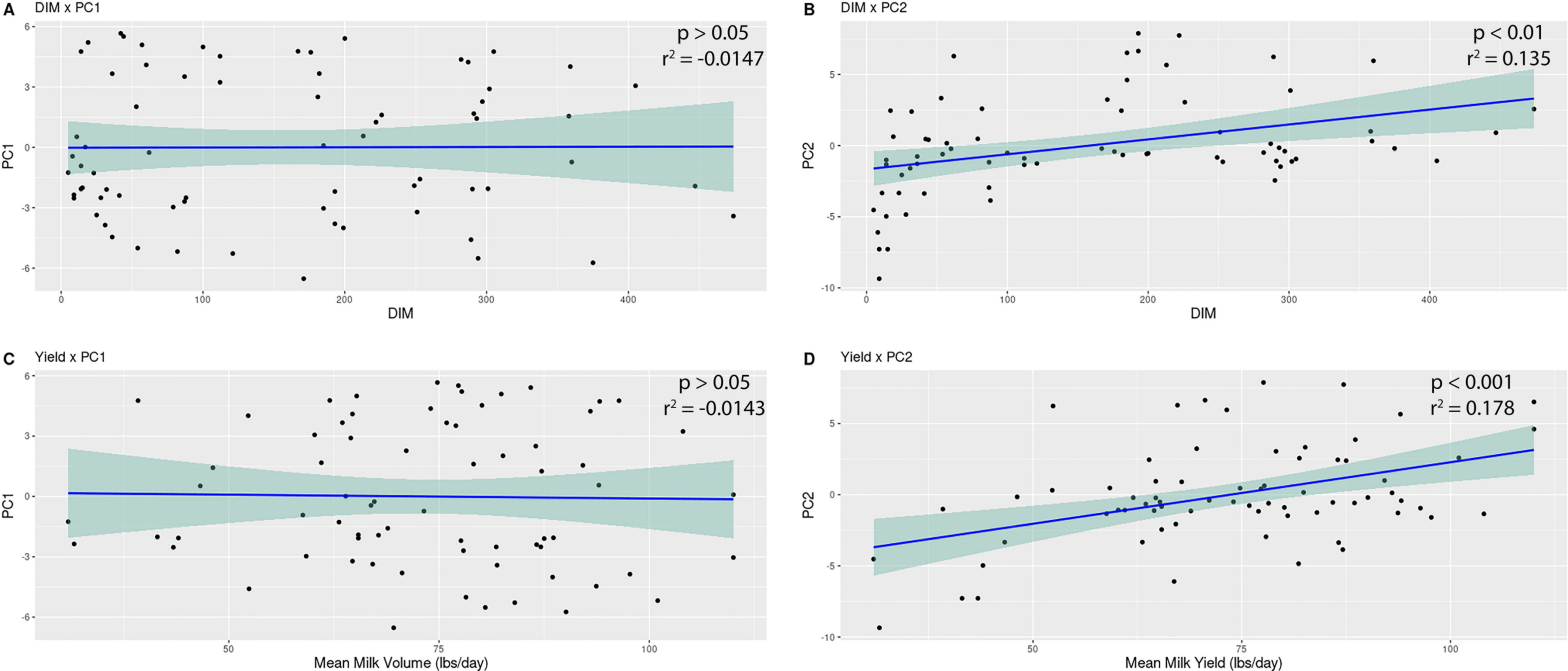
Linear modeling of days in milk (A and B) and milk yield (C and D) by PCA axes for first lactation animals. The location of the first lactation animals on the PCA axes was extracted and plotted against the animals’ respective days in milk or average milk yields. The lm() function in R was used to test for a significant relationship (*P* < 0.05) between the values.

Using the PCA we generated to document the istructure of the bacterial community, we tested for relationships between variables and the positions of the samples on the plot. The location of the samples on the PCA plot and either DIM or milk yield and the *X*- and *Y*-axis locations of all samples were extracted from the plot and used to perform a linear regression analysis against our variables of interest. In the first lactation, there was a significant relationship between both DIM and milk yield and the *Y*-axis of the PCA plot (*P* < 0.05; Fig. 2). In the second and third lactations, there was no significant relationship between DIM or milk yield and the PCA plot axes (*P* > 0.05; Fig. S4).

A DESeq2 analysis of differentially abundant ASVs associated with DIM and milk yield identified a number of ASVs (Table 1). When comparing all cows, regardless of parity, against DIM, 61 ASVs were identified, with a majority of these classified into the families *Prevotellaceae*, *Acidaminococcaceae*, and *Streptococcaceae*. A comparison of the first lactation animals identified 84 ASVs, with a majority classified into the aforementioned families. The second and third lactations identified one and two ASVs, respectively, classified to the families *Moraxellaceae*, *Spirosomaceae*, and *Haliangiaceae*.

**TABLE 1:** Significantly differentially abundant ASVs by days in milk or milk volume^*a*^

Phylum	Family	Genus	All lactation				First lactation				Second lactation				Third lactation			
			Low DIM	High DIM	Low milk weight	High milk weight	Low DIM	High DIM	Low milk weight	High milk weight	Low DIM	High DIM	Low milk weight	High milk weight	Low DIM	High DIM	Low milk weight	High milk weight
Actinobacteriota	*Actinomycetaceae*	*Actinomyces*				1												
	*Bifidobacteriaceae*	*Bifidobacterium*						1										
	*Bifidobacteriaceae*	Unclassified					1											
	*Corynebacteriaceae*	*Corynebacterium*						1										
	*Micrococcaceae*	*Garicola*						1										
		*Rothia*		2														
Bacteroidota	*Bacteroidaceae*	*Bacteroides*				1												
	*Flavobacteriaceae*	*Flavobacterium*	1			1												
		Unclassified						1										
	*Muribaculaceae*	Unclassified								1								
	*Porphyromonadaceae*	*Porphyromonas*		1		1												
	*Prevotellaceae*	*Prevotella*	13		5	11	8	7	1	9								
		*Prevotella_7*				2												
		*Prevotellaceae* NK3B31 group	2				1											
		*Prevotellaceae* UCG-001					1	1										
		*Prevotellaceae* UCG-004					1											
		Unclassified	4		1	4		1		1								
	Unclassified	Unclassified				1		1										
	*Rikenellaceae*	*Rikenellaceae* RC9 gut group								1								
	*Spirosomaceae*	*Spirosoma*													1			
	*Weeksellaceae*	*Bergeyella*		2			1	1										
		*Empedobacter*				1												
		Unclassified		2				1										
Fibrobacterota	*Fibrobacteraceae*	*Fibrobacter*	1				1											
Firmicutes	*Acidaminococcaceae*	*Succiniclasticum*	3			2	4	1										
	*Carnobacteriaceae*	*Jeotgalibaca*				1												
		Unclassified		1		1		1										
	*Lachnospiraceae*	*[Eubacterium] ventriosum* group						1										
		*[Ruminococcus] gauvreauii* group	1				1											
		*Acetitomaculum*	1				1											
		*Lachnospiraceae* NK3A20 group			1			1										
		*Moryella*					1											
		Unclassified	2	1	1	1	2	1										
	*Lactobacillaceae*	*Lactobacillus*		1				1										
		*Leuconostoc*				1												
		*Pediococcus*						2										
		*Weissella*				1												
	*Oscillospiraceae*	NK4A214 group					1	1										
	*Staphylococcaceae*	Unclassified						2										
	*Streptococcaceae*	*Streptococcus*		4	1	1		7										
		Unclassified		3	1			1										
	Unclassified	Unclassified		2		3		3		1								
Fusobacteriota	*Fusobacteriaceae*	*Fusobacterium*				1												
	*Leptotrichiaceae*	*Caviibacter*				1												
	*Leptotrichiaceae*	Unclassified						1										
Myxococcota	*Haliangiaceae*	*Haliangium*													1			
Patescibacteria	Unclassified	Unclassified		2	1			1										
Proteobacteria	*Burkholderiaceae*	*Lautropia*						1										
	*Cardiobacteriaceae*	*Suttonella*				1												
	*Moraxellaceae*	*Acinetobacter*				2								1				
		*Moraxella*		3	1			2										
		Unclassified						1				1						
	*Neisseriaceae*	*Alysiella*		1														
		*Conchiformibius*		1				1		1								
		*Neisseria*				1		1										
		Unclassified				2		3										
	*Oxalobacteraceae*	*Janthinobacterium*						1										
	*Pasteurellaceae*	*Aggregatibacter*						1										
		*Bibersteinia*			1			3										
		*Mannheimia*								1								
		Unclassified		4		1		3										
	*Rhizobiaceae*	*Neorhizobium*						1										
		Unclassified						1										
	*Succinivibrionaceae*	*Succinivibrionaceae* UCG-001				1												
	*Thiotrichaceae*	*Thiothrix*		1		1												
	Unclassified	Unclassified	1															
	*Synergistaceae*	*Pyramidobacter*	1															
Spirochaetota	*Spirochaetaceae*	*Treponema*				1												
Synergistota	*Synergistaceae*	*Pyramidobacter*					1											

^*a*^ DESeq2 was used to test for significantly differentially abundant ASVs when compared to the days in milk or average milk yields. This test was run for all animals and when split by lactation. Values represent the number of ASVs classified to that genus that had significantly higher relative abundances for the respective lactation group and metric.

A differential abundance analysis of ASVs associated with milk yield identified 59 ASVs for all animals, regardless of parity (Table 1). A majority of these were classified to the families *Prevotellaceae*, *Pasteurellaceae*, *Moraxellaceae*, and *Acidaminococcaceae*. Sixteen ASVs were identified in the first lactation group, classifying primarily to the family *Prevotellaceae*. One ASV, classified to the family *Moraxellaceae*, was differentially abundant in the second lactation, and no ASVs were found to be differentially abundant in the third lactation group. Full results of the DESeq2 analyses can be found in Table S3.

### Milk production efficiency is associated with the overall structure and specific members of the swab bacterial community

**Fig 3:**
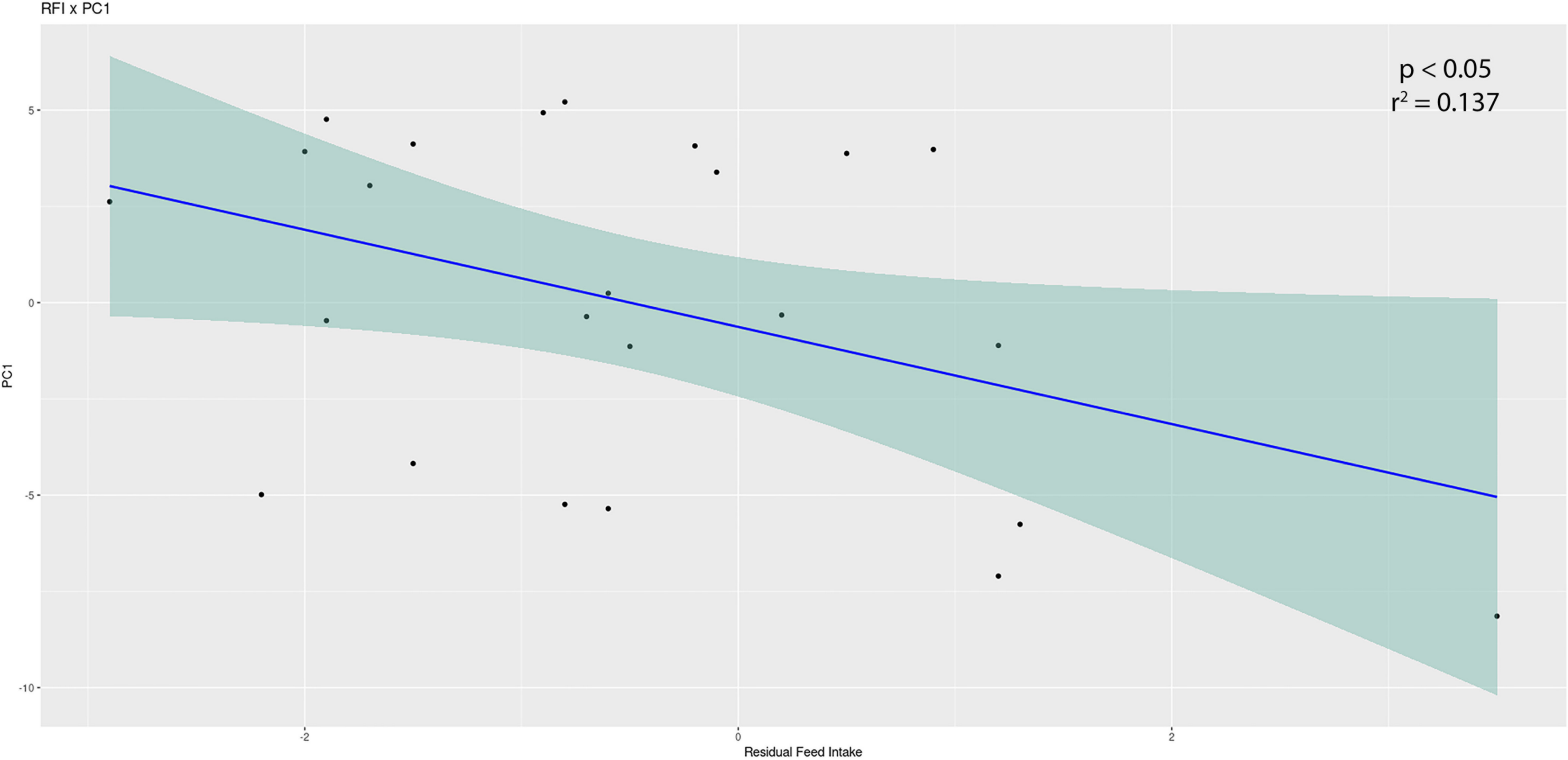
Linear modeling of residual feed intake by the PCA *X*-axis for second lactation animals. The location of the second lactation animals for which we have residual feed intake values was plotted against the *X*-axis of the PCA plot. The *lm*() function in R was used to test for a significant relationship between the values (*P* < 0.05).

Within our second and third lactation groups, we had a subset of animals for which milk production efficiency data were collected. In the second lactation group, when comparing alpha diversities between high- and low-efficiency animals (defined as the highest and lowest 20% GFE or RFI cows), there was a significant difference between high and low GFE groups and low and mid GFE groups (*P* = 0.046 and *P* = 0.010, respectively). In the RFI comparison, the low and mid groups were significantly different (*P* = 0.046) (Fig. S5A through F). PCA plots paired with a PERMANOVA indicated no significant differences in beta diversity between efficiency groups, but linear modeling of RFI with each sample’s positions on the *X*-axis of the PCA plot indicated a significant relationship between RFI and the structure of the communities (Fig. S6A and B; Fig. 3). DESeq2 analysis identified 130 ASVs associated with high GFE cows and 14 ASVs associated with low GFE cows, including genera common to the rumen such as *Prevotella*, *Fibrobacter*, *Succiniclasticum*, and *Butyrivibrio*. Oral-associated genera were also identified, including *Moraxella*, *Bibersteinia*, and *Neisseria*. We further identified 4 ASVs associated with high RFI animals and 101 ASVs associated with low RFI animals (Table 2).

**TABLE 2:** Significantly differentially abundant ASVs in second and third lactation animals divided by performance as calculated using gross feed efficiency of residual feed intake^*a*^

Genus	Second lactation				Third lactation			
	Low GFE	High GFE	Low RFI efficiency	High RFI efficiency	Low GFE	High GFE	High RFI (low efficiency)	Low RFI (high efficiency)
*Bifidobacterium*			2					
*Pseudoscardovia*			1					
*Brachybacterium*						1		
*Kocuria*						1		
Unclassified		1						
Unclassified		1						
Unclassified						1		
Unclassified		2						
*Prevotella*		61	38					
*Prevotellaceae* Ga6A1 group		1	1					
*Prevotellaceae* NK3B31 group		1	1					
*Prevotellaceae* UCG-001		6	4					
*Prevotellaceae* UCG-003		2						
*Prevotellaceae* YAB2003 group		1	1					
Unclassified		3	4					
Unclassified		2						
*Rikenellaceae* RC9 gut group		1	1					
*Bergeyella*						1		
*Fibrobacter*		1						
Unclassified			1					
*Succiniclasticum*		3	3					
*Aerococcus*		1						
*Facklamia*		1						
*Anaerovorax*		1						
*Christensenellaceae* R-7 group		1						
*Enterococcus*		1						
*Sharpea*			1					
Unclassified		1	1					
*Saccharofermentans*			2					
*[Eubacterium] ruminantium* group		4	4					
*Butyrivibrio*		3	3					
FD2005		1						
*Lachnospira*		1	2					
*Lachnospiraceae* AC2044 group		1	1					
*Lachnospiraceae* NK3A20 group		1	2					
*Moryella*		1	2					
*Oribacterium*			1					
Probable genus 10		1						
*Pseudobutyrivibrio*		2	2					
*Shuttleworthia*		1	2					
*Unclassified*		8	10					
*Companilactobacillus*	2							
*Levilactobacillus*	6							
Unclassified	3							
NK4A214 group		3	4					
UCG-005		1	1					
*Ruminococcus*		1						
Unclassified			1					
*Anaerovibrio*		1	1					
*Schwartzia*			1					
Unclassified			3					
*Veillonellaceae* UCG-001		1						
*Streptococcus*				4		2		
Unclassified						1		
Unclassified		1						
*Moraxella*	2					3		2
Unclassified								
*Neisseria*						1		
Unclassified						1		
*Janthinobacterium*						1		
*Bibersteinia*	1					2		
Unclassified						3		
*Pseudomonas*		1						1
*Sphingomonas*						1		
*Ruminobacter*		1						
*Succinivibrionaceae* UCG-002		1						
*Treponema*		1						
Unclassified		1						
Unclassified		1						

^*a*^ DESeq2 was used to test for significantly differentially abundant ASVs when compared to gross feed efficiency or residual feed intake scores. This test was run with second and third lactation animals for which there were GFE and RFI scores. Values represent the number of ASVs classified to that genus that had significantly higher relative abundances for the respective lactation group and metric.

For the third lactation group, there were no significant differences between groups of alpha diversity scores when comparing both GFE and RFI groups (*P* > 0.05; Fig. S5G through L). There was also no significant difference between efficiency groups when plotted against PCA (*P* > 0.05; Fig. S6C and D). Linear modeling of GFE against each sample’s position on the *X*-axis of the PCA plot indicated a significant relationship between GFE and the structure of the communities, and no significant relationship between RFI and PC2 (*P* < 0.05; *P* = 0.073) (Fig. S7A and B). DESeq2 analysis identified a higher relative abundance of 19 ASVs in the high GFE group and 3 ASVs in higher relative abundance in the low RFI (high efficiency) group (Table 2).

## DISCUSSION

Incorporating rumen microbial data into farm management has been a long-standing goal for the dairy industry, as it has the potential to improve milk production (11, 22, 23). However, developing such strategies is hindered by the inability to rapidly and easily sample the ruminal microbiome on a large scale, as traditional sampling methods are laborious and time-consuming. Recent work has shown that buccal swabbing can be an effective proxy for characterizing the ruminal bacterial community and has further been correlated with feed intake (21, 24). However, it is unknown if the bacterial community characterized from swabs can be correlated with other milk production metrics and if such associations remain across a large number of sampled animals. In this study, we sought to identify correlations between bacterial community characteristics and four different metrics for a cohort of 226 cows: days in milk, average milk yield, gross feed efficiency, and residual feed intake.

Previous work has established that the ruminal bacterial community is dynamic over time and between animals of differing production metrics (4). These earlier studies used samples collected directly from the rumen (e.g., stomach tubing and via cannula). Using buccal swabs, we found similar results, but not complete replication. For example, Jewell et al. (4) found an increase in rumen liquid and a decrease in rumen solid alpha diversity scores from the first to second lactation. Here, we found no significant differences in alpha diversity scores both within and between lactation groups (Fig. S1) (4). Alpha diversity is a quantification of the presence and/or abundance of species within samples. Our samples include a mix of rumen solid, rumen liquid, and oral bacteria, and the combination of ruminal fractions and potential exclusion of less abundant ruminal bacteria from our buccal swabs may impact our alpha diversity analyses and hinder our ability to identify differences between groups in different stages of lactation. We may be able to minimize this effect in the future by timing buccal swabbing strictly to when the cow is ruminating to ensure maximal capture of rumen contents or by using sequencing technology that captures a longer read length across the entirety of the 16S rRNA gene (e.g., Oxford Nanopore and Pacific Biosciences).

We then sought to identify which genera were highly correlated with days in milk and average milk yield. We identified both rumen- and oral-associated microbes that were significantly correlated with an increase in DIM (Fig. 1). Two unclassified genera within the families *Bifidobacteriaceae* and *Pseudomonadaceae* were negatively associated with DIM. Members of these families have previously been identified in the rumen and lower digestive tract of ruminants (11, 25). *Alysiella* and *Moraxella* were both positively correlated with DIM in our analysis. Previous research has shown that these genera are associated with the oral community (20). A fifth genus, *Conchiformibius* (formerly *Simonsiella*), was also positively correlated with DIM in our analysis. This genus was not identified in our previous work as oral-associated, but it is a member of the family *Neisseriaceae*, which contains a number of oral-associated microbes and has been identified from the oral microbiome of other animals (26). *Conchiformibius* has been found in the rumen of lambs, but it was most abundant in the 30 days after birth, when the rumen and its associated bacterial community are the least developed, and the oral community may have an outsized effect on the rumen community (27). Jami et al. (11) found a number of rumen-associated genera with strong correlations to milk yield, but in our data set, we were unable to identify any significant correlations between milk yield and the most abundant genera, which may be due to a number of factors, such as the inclusion of oral microbes in the data set, different rearing practices of the animals, and dietary differences.

Previous studies examining changes over lactations and between tiers of production metrics found differences in the overall structure of the bacterial communities (4, 11, 13). Because there was no significant difference between our categorical groups overall as plotted on the PCA plot (low-high milk yield), we attempted to identify significant correlations between both milk yield and DIM and the location of the samples on the PC1 and PC2 axes of the PCA plot. We identified significant correlations between both milk yield and DIM and the PC2 axis of the PCA plot for first lactation animals (Fig. 2B and D), similar to the results reported in Jewell et al. (4). This significant relationship indicates that beta-diversity is directly affected by milk yield and DIM.

Our differential abundance analysis identified numerous ASVs associated with DIM and milk yield (Table 1). A difficulty with interpreting these results is the level of taxonomic resolution we achieved with our amplicon data set. Many of the genera and families identified in our differential abundance analyses have members associated with high and low efficiency phenotypes, but it is challenging to determine the exact species and/or strains without more resolved classification such as through long-read sequencing.

The largest number of differentially abundant ASVs was identified in our first lactation group. Of these ASVs, a large proportion was classified as common rumen microbes (4, 12, 28, 29). *Prevotella* is a dominant genus within the rumen, and the main fermentative products of this genus include volatile fatty acids associated with both increased (acetate and butyrate) and decreased (propionate) milk production efficiency (30). *Ruminococcus* digests fiber, producing acetate (among other products), which has been associated with improved milk production (28, 31). *Succiniclasticum* was also identified as being differentially abundant. A main fermentative product of this genus is propionate, high levels of which are associated with reduced milk production efficiency (29, 31). These genera have been associated with both changes in milk production and stage of lactation (4, 11). We also identified ASVs in the genus *Pediococcus,* which has been isolated from other ruminants (32–35). One of its main fermentative products is lactic acid, and it has been investigated for its role in both the onset of ruminal acidosis and as a probiotic to improve efficiency and reduce methane production (32–35).

Within our herd, we had a subset of animals from the second and third lactations with previously collected milk production efficiency data. This included GFE and RFI, two different measurements of the ability of a cow to convert calories from feed into milk (4, 36–38). Previous studies using directly collected rumen samples have noted significant differences in the microbial characteristics of high- and low-efficiency animals (3, 4). In our second lactation animals, the high GFE cows displayed significantly higher Shannon’s diversity scores (Fig. S5A). While the high RFI efficiency group visually displayed lower scores overall, there was no significant difference between the high- and low-efficiency groups.

The significant correlations we observed between the position of samples on the *X*- and *Y*-axes of the PCA plots and the animal’s GFE and RFI scores in both the second and third lactations indicate that the structure of the microbial communities collected from the swabs is associated with production efficiency. These correlations further suggest that buccal swabs can be used to characterize the potential level of production of lactating cows (Fig. 3; Fig. S7). This partially mirrors the results reported by Jewell et al. (4), where they found a significant correlation between production efficiency metrics and rumen microbial community structure. We note that the study by Jewell et al. (4) focused on a smaller cohort of 22 animals and utilized a different sequencing technology (454 pyrosequencing) that targeted a different region of the 16S rRNA gene. However, given that we observed similar associations, regardless of sample type, methods, and sample size, our results confirm the validity of these and previous results.

The results of our differential abundance analyses were dominated by bacteria commonly found in the rumen and known to be associated with milk production efficiency (Table 2) (4, 11, 39). In our second lactation group, a majority of the ASVs identified as differentially abundant were in higher relative abundances in the high efficiency GFE group (130/144). Many of these are also common rumen bacteria, including those in the families *Prevotellaceae, Lachnospiraceae, Ruminococcaceae,* and *Succinivibrionaceae*, all of which have been previously associated with milk production efficiency (4). The *Lacnospiraceae* family ferments fibers to produce butyrate, which has been associated with improved milk production efficiency (40, 41). *Succinivibrionaceae* produces succinate, a precursor to propionate, high levels of which are associated with decreased milk production efficiency (42, 43).

In the RFI group, a majority of ASVs identified as differentially abundant were in higher relative abundances in the low efficiency RFI group (101/105). These included members of the families *Prevotellaceae*, *Lachnospiraceae*, and *Ruminococcaceae*. Previous research suggests that high-efficiency RFI animals have lower overall alpha diversity because the rumen community may be more specialized for digesting feed (44). Our findings of a majority of the ASVs having significantly higher relative abundances in the low efficiency RFI group would agree with this conclusion, as the higher abundance of many ASVs would increase alpha diversity in the low efficiency RFI group. We note, however, that there was no overlap between ASVs identified in these two comparisons. Similarly, Cox et al. (3) and Jewell et al. (4,4) both identified a number of operational taxonomic units significantly correlated with production metrics, including those in the *Prevotellaceae, Lachnospiraceae,* and *Ruminococcaceae* families. Jami et al. (11) also identified correlations between these families and production metrics.

For our third lactation animals, we only identified oral taxa as being differentially abundant between efficiency groups. Using direct rumen sampling, Weimer et al. (12) identified differentially abundant bacteria between high- and low-efficiency animals in their third lactation. Although we did not identify differentially abundant rumen bacteria, it is difficult to determine the cause. First, Weimer et al. (12) used three pairs of high-low efficiency animals, making a direct comparison with our study difficult. Second, as cows progress through lactation, farms will select for higher production and efficiency, potentially making higher lactation animals as a group more efficient (45). This could lead to difficulties with identifying differences between communities, as they may not be as disparate as in early lactations. Considering oral swabs are only a proxy for the ruminal community, more minute differences between animals may fall outside the detection limit of the swabs.

Although oral swabs are a promising approach for relating the ruminal microbiome to host production metrics, it is important to note that the oral swab microbiome includes oral microbes, which may present a confounding factor. Here, we identified numerous oral microbes significantly associated with our phenotypes of interest. As such, we cannot discount the role these oral-associated bacteria may play in determining the productive efficiency of a cow. Indeed, Edwards et al. (46) identified a number of oral-associated bacteria that are metabolically active under ruminal conditions, and Marcos et al. (21) found that oral swabs can be used to improve models predicting feed intake, even more so than directly collected rumen samples. They noted that biofilm formers in the oral community can serve as predictive markers for predicting feed intake, thereby potentially affecting milk production efficiency. Given that we were able to identify differences in alpha diversity, beta diversity, and in the relative abundances of ASVs attributed to the phenotypes in our data set, it is possible that the inclusion of oral microbial data may have strengthened our associations, although this remains to be tested.

Overall, our study demonstrates that oral swabs can be used as a proxy for the ruminal microbiome and can further be used to correlate against a variety of milk production phenotypes of interest to both researchers and producers. Many of the associations we found were previously identified in earlier studies using directly collected rumen samples (3, 4, 11). Given that characterizing the rumen microbial community on a wide scale is time- and cost-intensive, it is incredibly difficult to incorporate microbial data into production-scale herd management decisions. We propose that the use of oral swabs would solve this issue and open up other avenues of research. For example, the prediction of a cow’s productive ability through oral swabbing of heifers and cows in the early stages of lactation could inform producers on herd selection and dietary manipulations to improve overall farm production. Additionally, the application of oral swabs to capture the ruminal microbiome for integration into genome-wide association studies would further add an additional trait for host genotype selection. In sum, our data support the utility of oral swabs as a method for capturing the ruminal microbiome that can be used for large-scale studies of correlations against host production metrics.

## MATERIALS AND METHODS

### Sample and metadata collection

Animals were housed at a research farm in Arlington, WI, USA. All animals in the study were a part of the general milking herd kept in freestall housing. Diets were consistent for all animals except for animals in the first 55 days of any lactation, who received hay in addition to the total mixed ration (TMR) (1.5% of total TMR; Table S1). Sampling staff were trained in advance of sampling, and samples were collected between 15 and 16 October 2019. The methods for swab collection, sample preparation, and sequence cleanup are detailed in Skarlupka et al. (24,24) and Young et al. (20,24). In short, samples were collected before morning feeding, with the previous night’s feed having been removed approximately an hour before sampling. Puritan PurFlock flocked swabs (Puritan Medical Products, Guilford, ME, USA) were used to scrape the back cheek of the mouth of lactating cows for 15 s. Collected swabs were then placed into individual tubes containing 0.5 mL 1× phosphate-buffered saline, chilled on ice, and transported to the University of Wisconsin—Madison in Madison, WI, USA, for storage and further analysis.

Seven-day averages of daily milk yields (lbs/day) were calculated by the farm for animals in their first, second, or third lactation on the day of swab collection (*n* = 226). Data regarding the efficiency of converting consumed feed into converted milk energy represented a subset of the data described most recently in Cavani et al. (47), which are part of a larger global effort to improve feed utilization efficiency and reduce enteric methane emissions through genetic selection, as described in van Staaveren et al. (48). These studies collected milk production efficiency data from cohorts of animals on the farm at various times. This included milk energy, dry matter intake (DMI), metabolic body weight, change in body weight, and days in milk at the time of efficiency data collection. Residual feed intake was calculated as a part of the Cavani et al. (49) study from a regression, including these variables. RFI represents the difference between the observed and expected DMI, as determined by the previously mentioned regression. We interpreted lower RFI to generally indicate that the animal is more efficient at producing milk given the same feed and environment. To analyze differences between high- and low-efficiency animals and to minimize the effect of time and diet on the cow’s productive ability, we only used animals for which a milk production efficiency trial was started within 90 days of our sampling dates and had a collected oral swab (*n* = 43).

### Splitting by parity and determining high- and low-efficiency groups

All cows were first split into three groups based on their parity (number of lactations; 1: *n* = 74, 2: *n* = 79, and 3: *n* = 73). Due to the natural changes in milk production of individuals over the course of a lactation, cows were further split into stages of lactation as determined by days in milk at the time of swab collection: early (0–101 days), middle (102–202 days), and late (>202 days).

Within this group of 226 cows, we had a subset of animals for which we had milk production efficiency data. We subset these animals based on parity, but due to the low numbers of first lactation animals, we chose to only consider second and third parity animals (first lactation, *n =* 8; second lactation, *n* = 24; and third lactation, *n =* 15).

Within the second and third lactations, cows were assigned to high- and low-efficiency groups using two methods. The first approach calculated GFE, which was determined by dividing each cow’s milk energy (milkE) by DMI. The highest and lowest 20% of GFE scores were given high (HE) and low (LE) efficiency attributes, respectively. The middle range of cows was given the “MID” attribute.

The second method calculated RFI based on a regression of variables known to affect production: age, DIM, parity, cohort, and known metabolic energy sinks (36, 37). The regression was created using data from the USDA’s national herd database. A lower RFI suggested that the animal is a more efficient milk producer relative to the average cow. The highest and lowest 20% of RFI scores were given LE and HE attributes, respectively, with the remaining cows being given the “MID” attribute. It is important to note that GFE and RFI are two different methods of calculating efficiencies, so the animals in these groups differ. Production data, metadata, and GFE/RFI group assignments can be found in Table S2.

### DNA extraction, PCR amplification, and sequencing

Sample processing and sequence generation are detailed in Skarlupka et al. (24). In short, genomic DNA was extracted from the swabs using the Zymogen Quick-DNA HMW MagBead Kit (D6060: Zymo Research Corp., Irvine, CA, USA) adapted for a 96-well format. DNA was quantified using Qubit fluorometer reagents. The V4 region of the 16S rRNA gene was amplified using barcoded universal primers with adapters for sequencing on an Illumina MiSeq (V4 region primers: F- GTGCCAGCMGCCGCGGTAA, R- GGACTACHVGGGTWTCTAAT; Illumina Adapters: F-AATGATACGGCGACCACCGAGATCTACAC, R-CAAGCAGAAGACGGCATACGAGAT). Regions were amplified using a Bio-Rad S1000 thermocycler (Bio-Rad Laboratories, Hercules, CA, USA) with 2× KAPA HiFi HotStart ReadyMix (KAPA Biosystems; Wilmington, MA, USA) at 30 cycles of 95°C for 3 min, 55°C for 30 s, and 72°C for 30 s, ending with a final extension at 72°C for 5 min.

A 1% low-melt agarose gel (Gold Biotechnology; St. Louis, MO, USA) was used to confirm successful amplification and purify PCR products. DNA was extracted from the gel pieces, and samples were equimolarly pooled to 4 nM. The pooled libraries were sequenced using an Illumina MiSeq v2 2 × 250 kit (Illumina; San Diego, CA, USA) at 11 pmol/L with 10% PhiX control.

### Sequence cleanup

The resulting sequencing files were cleaned, and ASVs were generated using DADA2 (version 1.24.0) in R (version 4.2.1) (50, 51). To summarize, the reads were filtered and trimmed before taxonomy was assigned using the Silva reference database (version 138.1), with species-level assignments made using the same database (52). A phyloseq object was created using the R package phyloseq (version 1.48.0), and non-bacterial sequences were removed (53). Rarefaction curves were used to visually determine the number of sequences needed to achieve sufficient sequencing depth. These curves indicated that 7,000 sequences effectively captured the bacterial community while minimizing the removal of samples due to low sequence counts. Samples with fewer than 7,000 sequences were removed, and an abundance cutoff was applied where any ASV with fewer than 10 counts in every sample was removed. For alpha diversity and correlation analyses, samples were rarefied to 7,000 sequences prior to analysis.

### Statistical analyses

The estimate_richness() command in phyloseq was used to calculate alpha diversity values. Normality was tested for using shapiro.test() in the stats package (version 4.4.1) (50). For non-normal data sets, the Kruskal-Wallis test was used, followed by the Wilcoxon rank sum test for pairwise comparisons of significant results (*P* < 0.05) [kruskal.test(); pairwise.wilcox.test()]. Spearman’s rank correlation was used to determine if there was a relationship between alpha diversity scores and the 7-day average of milk yields [cor.test(…, method=“spearman”)].

To find genera associated with DIM and milk yield, a “core” list of genera was calculated with the following parameters: >0.1% abundance in at least one animal and >50% prevalence across all samples. Of the remaining genera, the 75 most abundant genera were used to calculate correlations with DIM and milk yield as determined by Spearman’s rank correlation with false discovery rate correction. Heatmaps showing positive and negative correlations were plotted using the ggplot2 package (version 3.5.1) (54).

A CLR transformation of the sequence counts was performed using the transform(…, “clr”) command from the microbiome (version 1.26.0) package (55). Principal component analysis plots were generated using Aitchison distances using the ordinate(…, “RDA”) function (53). This function generates a plot that can be used to visualize each sample’s overall microbial community relative to the other communities. Differences in beta diversity between groups were tested using PERMANOVA in the vegan package (version 2.6-6.1) [adonis2()] (56). To test if the location on the *X*- and *Y*-axes of the samples on the PCA plot could be correlated to variables of interest, the *X*-axis (PC1) and *Y*-axis (PC2) values were extracted, and the lm() function was used to perform linear modeling of the PC1 and PC2 values with our variables of interest (DIM and milk yield). Models were plotted with a 95% confidence interval. Our previous work has shown that the color of the oral swab has an impact on the structure of the bacterial community (24). We included this variable in our modeling using the lm() function and did not find any significant interaction between swab darkness and our variables of interest as they related to PC1 and PC2 (*P* > 0.05). Animals were further subset into groups based on parity using the above analyses.

The package DESeq2 (version 1.36.0) was used to identify ASVs that were significantly differentially abundant across the range of milk yield and DIM values (*P* < 0.05) (57). The phyloseq object was converted to a format usable by the DESeq2 package using phyloseq_to_deseq2(), and the differential abundance analysis was then performed using the function DESeq().

A comparison of alpha diversity scores between high and low RFI and GFE groups was made, followed by a beta diversity analysis as described above. Linear modeling of the samples’ RFI/GFE scores and locations on the *X*- and *Y*-axes was performed, and DESeq2 was used to identify microbes that were differentially abundant between the high and low GFE and RFI groups.

## Data Availability

Raw reads can be found on the NCBI’s Short Read Archive under BioProject number PRJNA1117920. R code, metadata, and phyloseq objects for data analysis can be found at https://github.com/JSkar/Swab_MilkProduction.

## SUPPLEMENTAL MATERIAL

Supplemental Figures msphere.00167-25-s0001.docx Figures S1 to S7.

Table S1 msphere.00167-25-s0002.xlsx Ration mixes for cows in the study.

Table S2 msphere.00167-25-s0003.csv Metadata, production data, and efficiency groupings for the cows in the study.

Table S3 msphere.00167-25-s0004.xlsx Full DESeq2 results for all analyses.

ASM does not own the copyrights to Supplemental Material that may be linked to, or accessed through, an article. The authors have granted ASM a non-exclusive, world-wide license to publish the Supplemental Material files. Please contact the corresponding author directly for reuse.
