# Supplementary material for: Correlating the oral swab microbial community with milk production metrics in Holstein dairy cows
Source: mSphere. 2025 May 14;10(6):e00167-25. doi: 10.1128/msphere.00167-25 (PMC12188706; doi:10.1128/msphere.00167-25)
Supplement: Supplemental Figures — Figures S1 to S7. [file msphere.00167-25-s0001.docx]

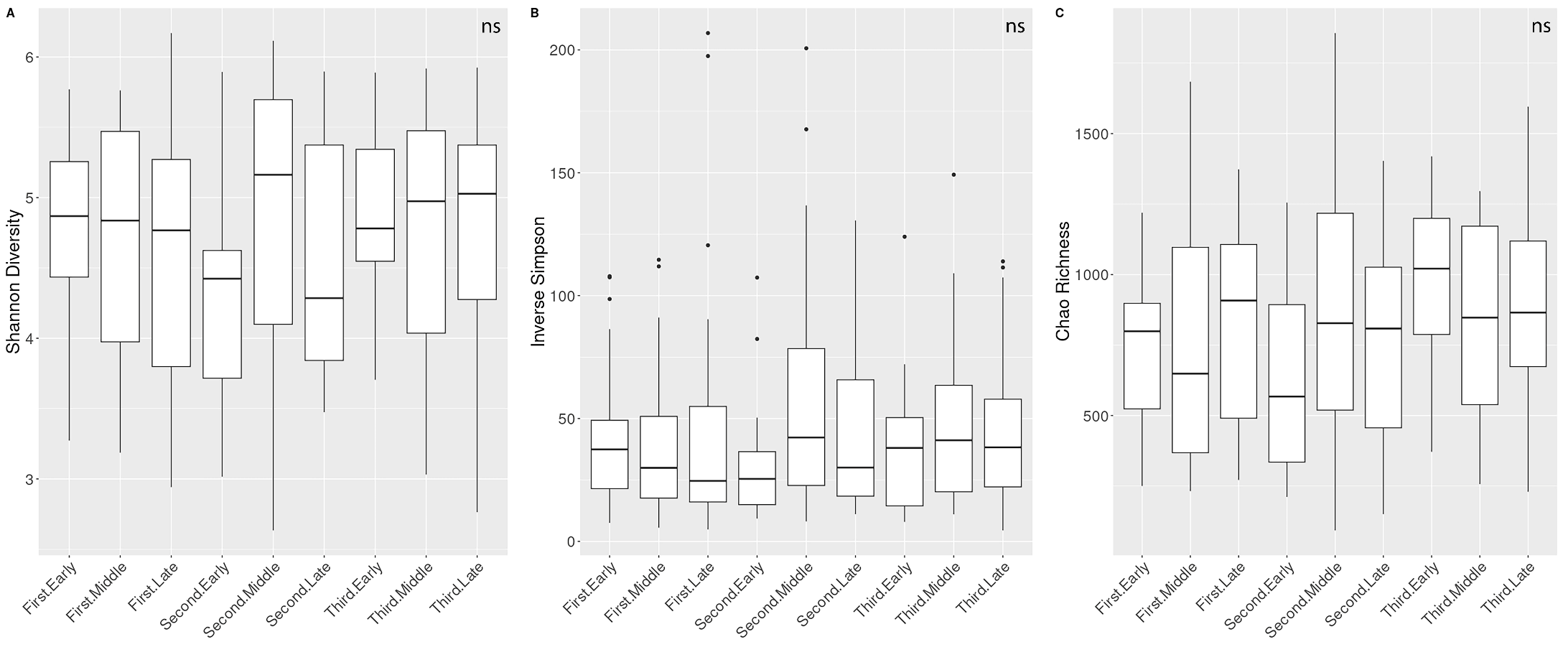


Supplementary Figure 1: Alpha diversities of animals grouped by lactation and stage of lactation.


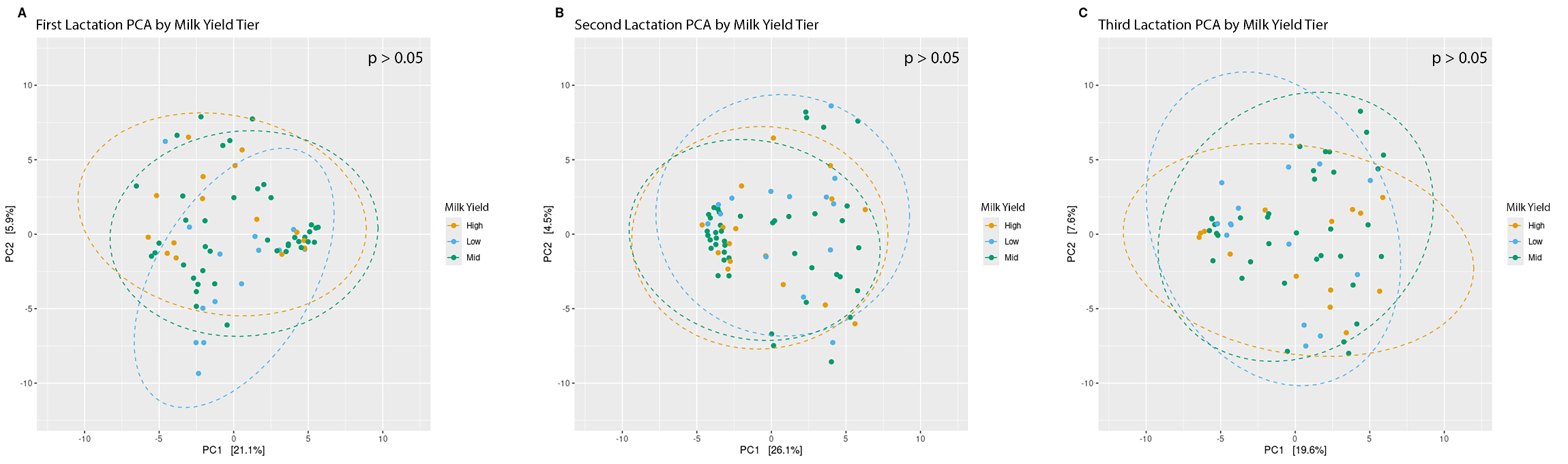
Supplementary Figure 2: PCAs of animals grouped by lactation and milk yield tier.


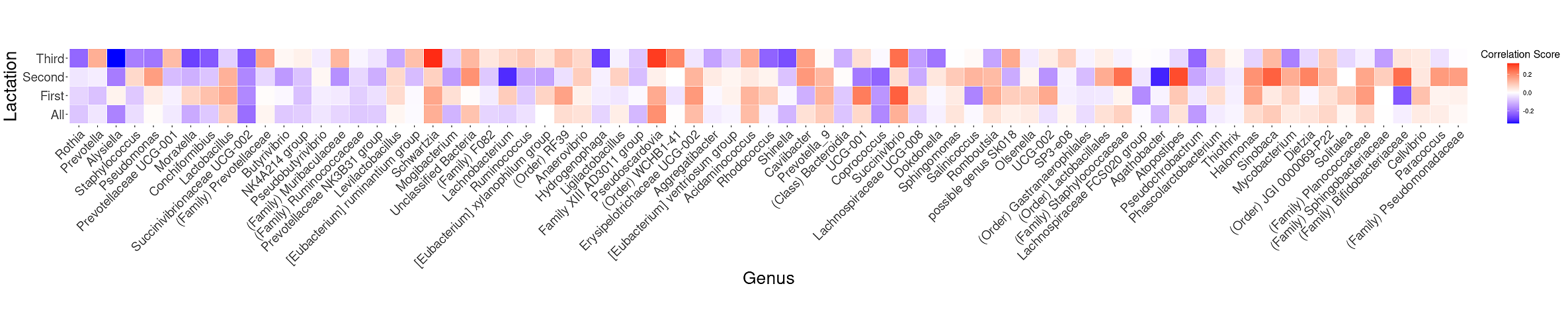


Supplementary Figure 3: Spearman’s correlation heatmap of the top 75 genera to average milk yield.


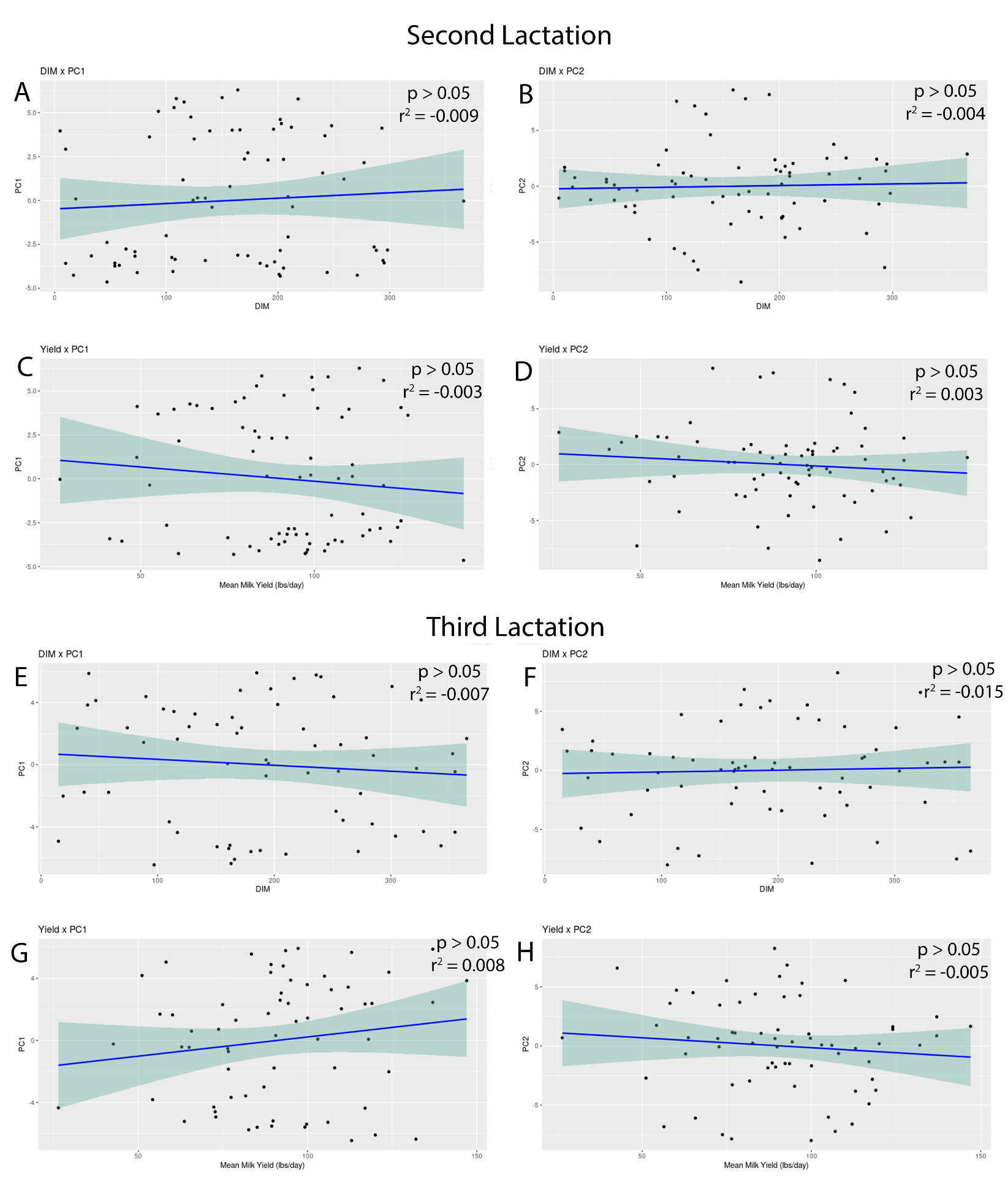
Supplementary Figure 4: Linear modelling of days in milk and milk yields of second and third lactation animals by their locations on the PCA X- and Y-axes.


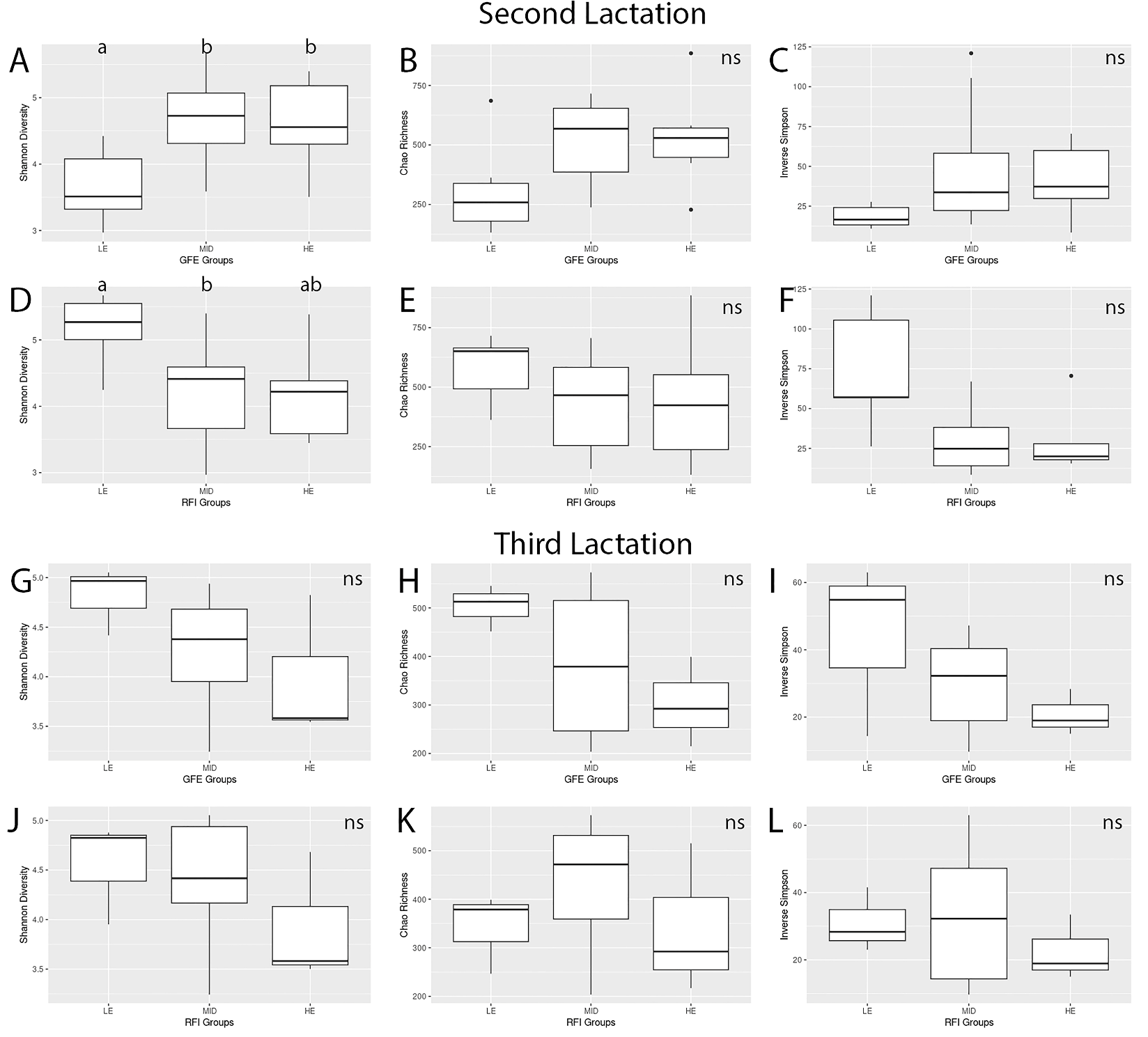


Supplementary Figure 5: Alpha diversities of second and third lactation animals grouped by GFE or RFI efficiency groups.


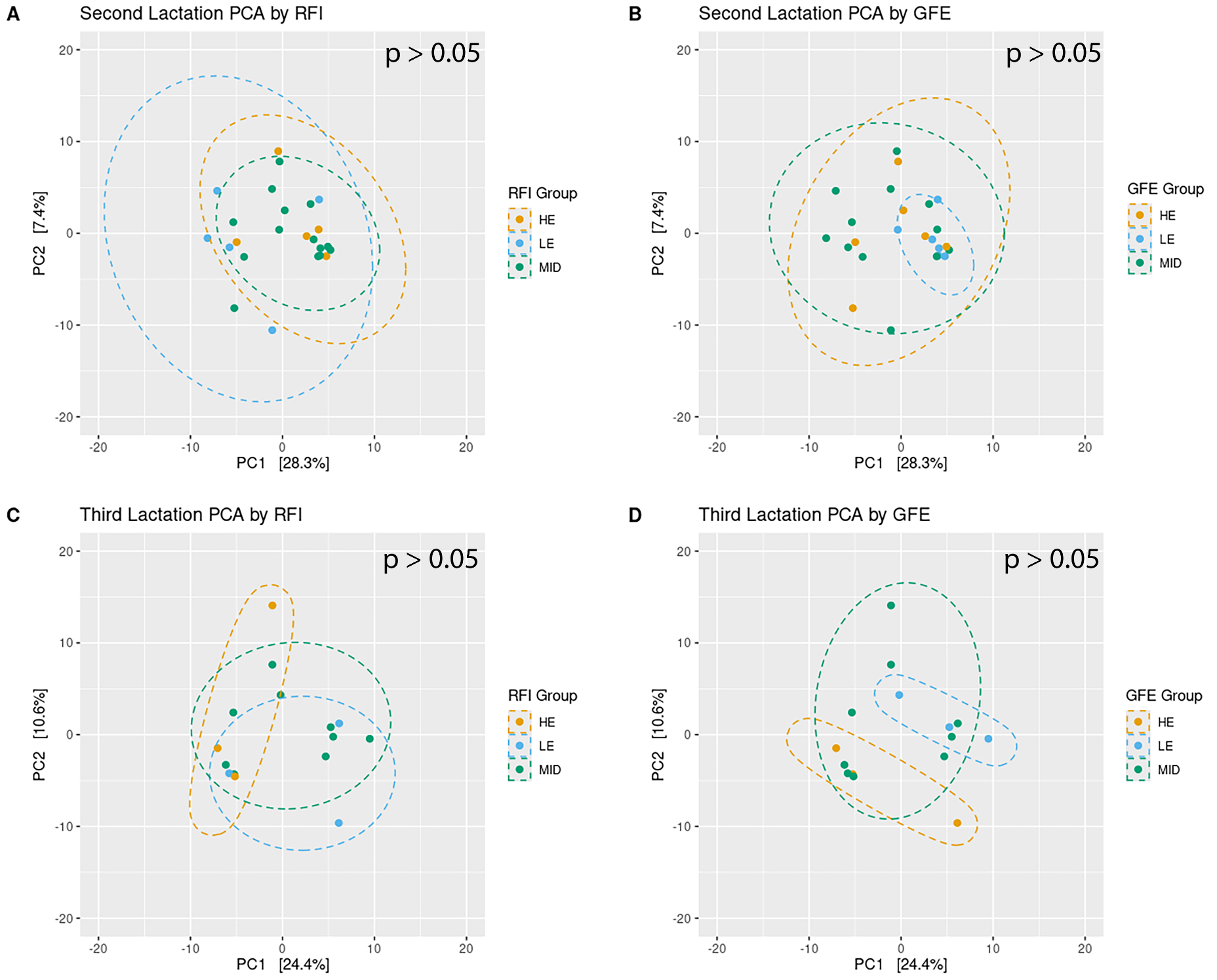
Supplementary Figure 6: PCAs of second and third animals grouped by GFE and RFI.


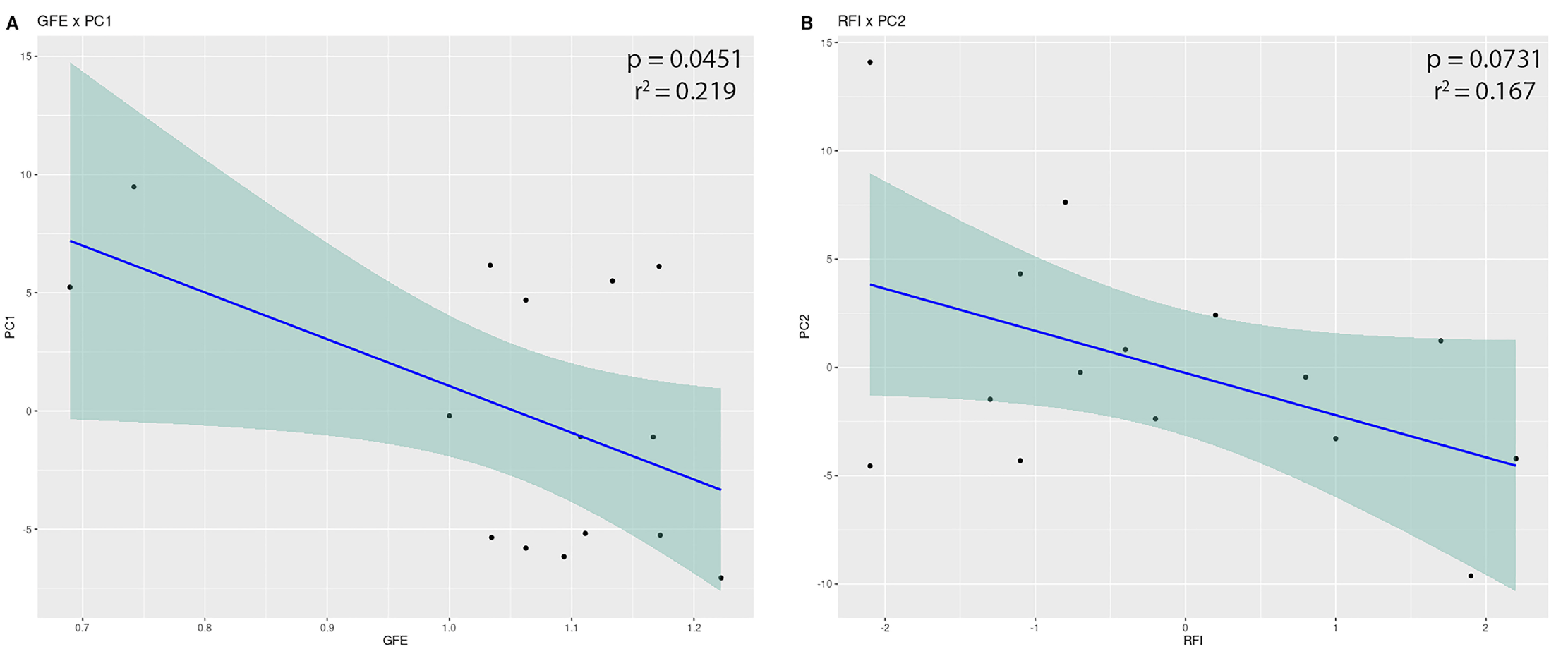


Supplementary Figure 7: Linear modelling of GFE x PC1 and RFI x PC2 for the third lactation animals.

Supplementary Table 1: Ration mixes for cows in the study.

Supplementary Table 2: Metadata, production data, and efficiency groupings for the cows in the study.

Supplementary Table 3: Full DESeq2 results for all analyses.
